# State-of-the-Art Cancer Immunotherapies

**DOI:** 10.3390/ijms25052532

**Published:** 2024-02-22

**Authors:** Hisashi Nagase, Takuma Kato, Takayuki Yoshimoto

**Affiliations:** 1 Department of Parasitology, Shinshu University School of Medicine 3-1-1, Asahi, Matsumoto 390-8621, Japan; hnagase@shinshu-u.ac.jp; 2 Department of Cellular and Molecular Immunology, Mie University Graduate School of Medicine, 2-174 Edobashi, Tsu 514-8507, Japan; katotaku@doc.medic.mie-u.ac.jp; 3 Department of Immunology, Nagoya University Graduate School of Medicine, 65 Tsurumai-cho, Showa-ku, Nagoya 466-8550, Japan; 4 Department of Immunoregulation, Institute of Medical Science, Tokyo Medical University, 6-1-1 Shinjuku, Shinjuku-ku, Tokyo 160-8402, Japan

Cancer immunotherapy is a type of cancer therapy utilizing the immune system to fight against tumors. In recent years, adoptive cell transfer (ACT) of chimeric antigen receptor (CAR)-T cells [1] and immune checkpoint inhibitors (ICIs) such as antibodies against programmed cell death 1 (PD-1) [2] has made remarkable progress. In addition, the mRNA vaccine delivered by lipid nanoparticles (LNPs) has successfully saved numerous lives from the coronavirus disease 2019 (COVID-19) [3], and therefore is currently expanding the application field to other diseases including cancers. Cytokines and exosomes are secreted mediators critical for cell-to-cell communication in the normal and tumor microenvironment. Several cytokines have been shown to exert antitumor activities in many preclinical models, and recent cancer immunotherapy with engineered cytokines targeting tumors with prolonged half-life is more effective [4]. Exosomes are a new type of extracellular vesicles that modulate the cell signaling pathway in normal and tumor cells via mainly microRNAs (miRNAs) [5]. This editorial briefly summarizes these latest advances in research on cancer immunotherapy ranging from basic research to clinical aspects that deepen our understanding of human cancer to eradicate it.

CAR-T cell therapy is a new type of ACT therapy and utilizes autologous T cells containing αβTCR (αβ T cells), which are engineered to express a CAR that targets specific antigens [1]. So far, six CAR-T therapies have been approved by the Food and Drug Administration (FAD) for the treatment of hematological malignancies including lymphomas, leukemia, and multiple myeloma. However, clinical trials of CAR-T therapy for patients with solid tumors have not succeeded yet because of limited infiltration into tumors. Cytokine release storm and immune effector cell-associated neurotoxicity are also potential drawbacks for CAR-T cell therapy. In addition, there are several concerns including time, logistics, cost of manufacturing processes, and exhaustion of T cells in heavily pretreated patients. To overcome these problems in autologous settings, allogeneic CAR-T, which is prepared from a third-party donor in advance with antitumor activity but not allogenic response, would be desirable [6]. To meet this criteria, CAR-T cells were prepared from Vγ9Vδ2 T (CAR-γδ T) cells, whose recognition is independent of the major histocompatibility complex (MHC) but dependent on butyrophilin 3A1/2A1 [7]. These CAR-γδ T cells can be expanded by using a novel prodrug of bisphosphonate and showed antitumor activity in an antigen specific manner. Thus, CAR-γδ T cells are a possible source of ‘off-the-shelf’ CAR-T cells for allogeneic ACT therapy. Similarly, natural killer (NK) cells are also possible alternative candidates for allogenic ACT therapy of CAR-T cells because they are also independent of MHC recognition and have a low risk of graft-versus-host disease [8]. Indeed, several CAR-NK cells targeting different tumor antigens have been prepared and studied in preclinical models. To introduce CAR gene into lymphocytes, there are several viral and non-viral delivery systems. Viral vectors are efficient but have several concerns associated with regulatory requirements, safety, and high cost, while non-viral methods such as DNR/RNA transfection have limitations in the efficiency of transfection into cells. The delivery of COVID-19 mRNA vaccines manufactured by Moderna [9] and Pfizer [3] was successfully achieved using LNPs. This non-viral delivery system has several advantages, such as high efficiency, high stability, and low toxicity. The LNPs efficiently delivered CAR mRNA into NK cells that were expanded from primary peripheral blood mononuclear cells using K562 feeder cells expressing 4-1BB ligand and membrane-bound interleukin (IL)-21 [10]. The resultant CAR-NK cells were shown to secret a high level of interferon (INF)-γ and granzyme B, kill multiple myeloma cells, and suppress the tumor growth of Nalm-6 leukemia in vivo [11]. Thus, CAR-NK cells also provide a possible candidate for ‘off-the-shelf’ CAR-T cells, and the mRNA-LNP technology is useful for gene introduction into cells.

Co-inhibitory receptors expressed on T cells, including PD-1 and cytotoxic T lymphocyte antigen 4 (CTLA-4), negatively regulate T cell-mediated immune responses. However, tumor cells exploit these inhibitory molecules to induce T cell exhaustion and tumor tolerance. Therefore, ICIs such as blocking antibodies against CTLA-4, PD-1, and PD ligand 1 (PD-L1) can be utilized to reactivate the immune response against tumor cells, and therefore have been approved by the FAD for the treatment of various types of cancer [2]. However, only a small sub-population of patients (20~40%) benefit from this therapy, suggesting the necessity to identify predictive biomarkers. Therefore, it is highly required to identify reliable biomarkers such as related molecules in the tumor microenvironment, microbiome, hypoxia, extracellular matrix, cytokines, and tumor mutational burden [12]. Indeed, the therapeutic efficacy of nivolumab (anti-PD-1) in gastric cancer was seen only in a limited number of patients, but patients who do respond often achieve long-term survival. To assess the intratumoral immune response in cancer patients, RNA sequencing data (called an immunogram) were established and successfully applied together with flow cytometry analysis to a cohort of gastric cancer surgery cases, resulting in classification into four distinct groups. A similar approach was applied to gastric cancer patients before nivolumab therapy and during early-on treatment with the drug, identifying the linkage of high T cell/regulatory T cell ratios and a low UV-radiation-response gene signature to durable clinical benefits [13]. Because ICI regulates the immunosuppressive responses by blocking the inhibitory signals, its treatment may also augment inflammatory and autoimmune responses, leading to adverse events in the skin, gastrointestine, nerves, and heart [14]. Indeed, approximately, one third of patients treated with ICIs develop adverse events including colitis, and in severe cases, ICI treatment might need to be discontinued. ICI-induced colitis is one of the leading causes of discontinuation and shows similar pathogenesis to inflammatory bowel disease, and therefore anti-TNF-α treatment is often offered to these patients [15].

Worldwide success of mRNA vaccination against severe acute respiratory syndrome coronavirus 2 (SARS-CoV-2) has saved numerous lives from COVID-19 [16]. Generally, intramuscular injection is easier, safer, and well tolerated to perform on a large scale, whereas intradermal injection is considered superior for the induction of protective immunity, but requires greater proficiency for the injection. To improve these issues, several different types of versatile jet injectors have been developed to deliver DNAs, mRNAs, proteins or drugs [17]. Among them, a new needle-free pyro-drive jet injector, which has a unique characteristic utilizing gunpower as a mechanical driving force, provokes high jet velocity and consequently the wide dispersion of the injected DNA solution in the skin. A number of studies revealed that it is highly effective as a vaccinating tool to induce potent protective cellular and humoral immunity against infectious diseases and cancers [18]. To enhance immunogenicity, COVID-19 mRNA is encapsulated in LNPs, and the resultant mRNA-LNPs nanoparticles migrate systemically and are broadly distributed in the human body after intramuscular injection. This strategy is effective for vaccination, but may also exert proinflammatory responses, leading to the development of adverse effects such as acute myocardial infraction, Guillan–Barr syndrome, and stroke. Of note, in place of LNPs, the needle-free pyro-drive jet injector might be utilized for intradermal injection of COVID-19 mRNA vaccines to reduce systemic adverse effects [19].

Cytokines and exosomes are secreted mediators critical for cell-to-cell communication in the normal and tumor microenvironment. Although potent antitumor activities of several cytokines such as IL-2, IL-12, IL-15, IL-18, IL-21, IL-27, granulocyte macrophage-colony stimulating factor, and IFN-α/β/γ have been demonstrated in many preclinical models, only limited success in clinical trials of IL-2 and IFN-α has been achieved [20]. Cytokine-based cancer immunotherapy is still promising, but these cytokine therapies have currently been supplanted by more efficacious therapies such as ICI. Nevertheless, cancer immunotherapy with engineered cytokines, i.e., cytokines combined with other immunotherapies to improve the targeting potential, half-life, and adverse effects, revives the potential of cytokine-based cancer immunotherapy [4]. Indeed, a novel IL-15-based immunocytokine called GT-00AxIL15, which includes antibodies targeting a tumor-associated glycosylated epitope of MUC1, has recently been developed and effectively delivered to solid tumors with potent antitumor effects [21]. Exosomes are extracellular vesicles that are secreted by cells and play a variety of physiological and pathological roles via cell-to-cell communication [5]. Exosomes function as drug delivery vehicles due to their unique properties of low immunogenicity, accumulation in tumors, and penetration capacity into distance organs. Exosomes contain various cargos such as proteins, lipids, and nucleic acids including mRNAs, noncoding RNAs, and DNA. These bioactive substances can be transferred into the target cells to change the transcriptome and modulate tumor-related signaling pathways. The effects of exosomes on tumor growth and metastasis highly depend on the source of cells and their contexts of pro- and anti-inflammatory situations. Interestingly, exosomes derived from anaplastic cells, such as the mouse melanoma cell line overexpressing Nang or iPS cells, were shown to exert higher suppressive effects on metastasis possibly via certain miRNAs [22].
